# Value of SPECT-CT Imaging for Middle-Aged Patients with Chronic Anterior Knee Pain

**DOI:** 10.1186/s12891-015-0628-9

**Published:** 2015-07-26

**Authors:** Du Hyun Ro, Ho-Young Lee, Chong Bum Chang, Seung-Baik Kang

**Affiliations:** Department of Orthopaedic Surgery, Seoul National University College of Medicine, Seoul, South Korea; Department of Orthopaedic Surgery, SMG-SNU Boramae Medical Center, Seoul, South Korea; Department of Nuclear Medicine, Seoul National University Bundang Hospital, Seongnam-si, South Korea

**Keywords:** Anterior knee pain, SPECT-CT, Patellofemoral arthritis, Chondral lesion, Cartilage, bone

## Abstract

**Background:**

Single-photon emission computed tomography-computed tomography (SPECT-CT) is a highly sensitive tool for detecting bone metabolism. We determined whether subchondral bone metabolism, as indicated by SPECT-CT in the patellofemoral (PF) joint, predicts response to conservative management in middle-aged patients with chronic anterior knee pain (AKP), and whether SPECT-CT results correlate with arthroscopic assessments of chondral lesions in the PF joint.

**Methods:**

The study group comprised 74 middle-aged patients with chronic AKP. All of the patients underwent SPECT-CT, and the results were graded along a scale of 0 to 3°. After 8 weeks of conservative management, they were grouped as responders (n = 40) or non-responders (n = 34) according to symptom improvement. We compared the median scintigraphic uptake of the PF joint between the two groups, and evaluated the positive predictive value (PPV) of uptake for treatment response in each patient. In non-responders, cartilage condition was assessed during arthroscopy, and the correlation of scintigraphic uptake with severity of the chondral lesion was assessed.

**Results:**

The median scintigraphic uptake in the patella was higher in non-responders than in responders (2 *vs.* 1). Among patients with higher patella uptake (grade 2 or 3), the PPV for non-response to conservative therapy was 62–67 %, whereas it was 24–25 % in patients with lower uptake (grade 0 or 1). Patella uptake corresponded strongly with arthroscopic assessment of patellar chondral lesions; the correlation was less strong for the femoral trochlea.

**Conclusions:**

Increased subchondral bone metabolism in the patella is associated with responsiveness to conservative therapy. SPECT-CT can benefit clinicians by predicting the treatment response from conservative management.

## Background

Anterior knee pain, or anterior knee pain syndrome, is one of the most common orthopedic disorders [1–4]. This disease entity has also been referred to as chondromalacia patellae (previously) and patellofemoral (PF) pain syndrome (more recently) [1–3, 5, 6]. Anterior knee pain has several causes including medial patellar plica, supra- and infra-patellar fat pad lesions, patellar and/or quadriceps tendinopathy, PF malalignment, and chondral lesions of the PF joint [1–3, 5, 6]. As these lesions can be objectively documented by imaging, particularly by magnetic resonance imaging (MRI), the lesion causing a particular patient’s knee pain may be identified [2, 7]. However, although several imaging modalities can reveal anatomical lesions related to the PF joint, such lesions, particularly chondral lesions in middle-aged patients, might not always be the source of anterior knee pain [2–4, 7, 8]. In fact, several studies have reported that chondral lesions identified by imaging modalities, or even during arthroscopy, do not correlate well with the presence or severity of knee symptoms in patients or the general population [7, 9].

Functional images generated by the administration of radioactive tracers, which map metabolic changes, can also assess a variety of orthopedic problems [10–17]. In particular, single-photon emission computed tomography-computed tomography (SPECT-CT), which is a combination of SPECT functional images with CT anatomical images, may overcome the aforementioned limitations of common anatomical imaging modalities for managing middle-aged patients with chronic anterior knee pain. SPECT-CT is very sensitive to changes in osteoblastic metabolism; metabolic changes indicate stress in the subchondral bone, which is related to pain [10, 12, 15, 16]. Recently, this technique has received attention as a means of assessing various knee problems including anterior knee pain. The intensity and distribution of metabolic tracer uptake on SPECT-CT correlates with patella height and tilt [17], and provides anatomical and physiological information, whereas other modalities such as radiographs, MRI, and conventional CT do not provide such information [16]. SPECT-CT may provide valuable prognostic information, and allow identification of the lesion causing anterior knee pain [12, 16, 17]. Nevertheless, limited information is available regarding the value of SPECT-CT for middle-aged patients with chronic anterior knee pain. Therefore, the primary purpose of this study was to investigate whether the intensity of subchondral bone tracer uptake in the PF joint predicts response to conservative treatment in middle-aged patients with chronic anterior knee pain. The secondary purpose was to determine whether arthroscopic findings of chondral lesions in the PF joint correlate with the intensity of bone tracer uptake.

## Methods

### Study subjects

We retrospectively reviewed a prospectively collected database of 151 patients with anterior knee pain who presented at our outpatient clinic from August 2009 to December 2011. All of the patients provided informed consent regarding the use of their medical records. Patients were selected based on the following criteria: age between 40 and 65 years, lack of traumatic cause of knee pain, and pain lasting at least 8 weeks. Patients presenting with a chief complaint of anterior knee pain were evaluated by radiography including a standing anteroposterior (AP) knee radiograph, a standing 45° flexion posteroanterior radiograph (Rosenberg view), 30° lateral knee radiographs, a Merchant PF radiograph, and a standing full-limb radiograph. The radiographic presence and degree of radiographic knee osteoarthritis (OA), as well as the presence of tibiofemoral (TF) and PF joint malalignments were assessed; sulcus angle, congruence angle, and Insall-Salvati ratio were also measured. In addition, patients underwent routine blood tests including uric acid, C-reactive protein (CRP), and erythrocyte sedimentation rate (ESR) to exclude the possibility of inflammatory knee arthritis. A single investigator (SBK) also took a detailed history and performed a comprehensive physical examination to determine the presence of previous trauma or other predisposing factors for anterior knee pain, as well as the presence of ligament injuries and/or meniscal injuries.

Of the 151 patients, 77 patients were excluded for the following reasons: (1) radiographic evaluation yielding Kellgren-Lawrence OA grade ≥ 2 in the PF and/or TF joints (n = 31); (2) history of knee, spine, or hip surgery (n = 17); (3) pain in the affected leg other than anterior knee pain, such as radiating pain (n = 5); (4) any form of knee instability on physical examination, a positive McMurray test, or a limited range of motion (n = 10); (5) any evidence of inflammatory knee arthritis such as gout or rheumatoid arthritis (n = 4); (6) evidence of habitual or recurrent patellar instability on physical examination, such as the apprehension test, passive patella translation test, J-sign test, and patella tilt test (n = 10). In total, 74 middle-aged patients remained with unilateral, atraumatic, and isolated anterior knee pain. The study population included 51 female and 23 male patients with a mean age of 54 years (standard deviation, SD: 6.1; range: 41–65) and a mean body mass index (BMI) of 26.3 kg/m^2^ (SD: 4.1; range: 18–36). The average sulcus angle was 129.7° (SD: 6.9; range: 112–149°), the average congruence angle was −4.4° (SD: 8.8; range: −25° to 14°), the Insall-Salvati ratio was 0.97 (SD: 0.12; range: 0.71–1.4), and the average Q angle was 9.9 ° (SD: 3.8; range: 0–22 °). All of the study protocols were approved by the institutional review board of our hospital (Seoul Metropolitan Government Boramae Hospital, Protocol No. 06–2012–153).

### SPECT-CT evaluation

**Fig. 1:**
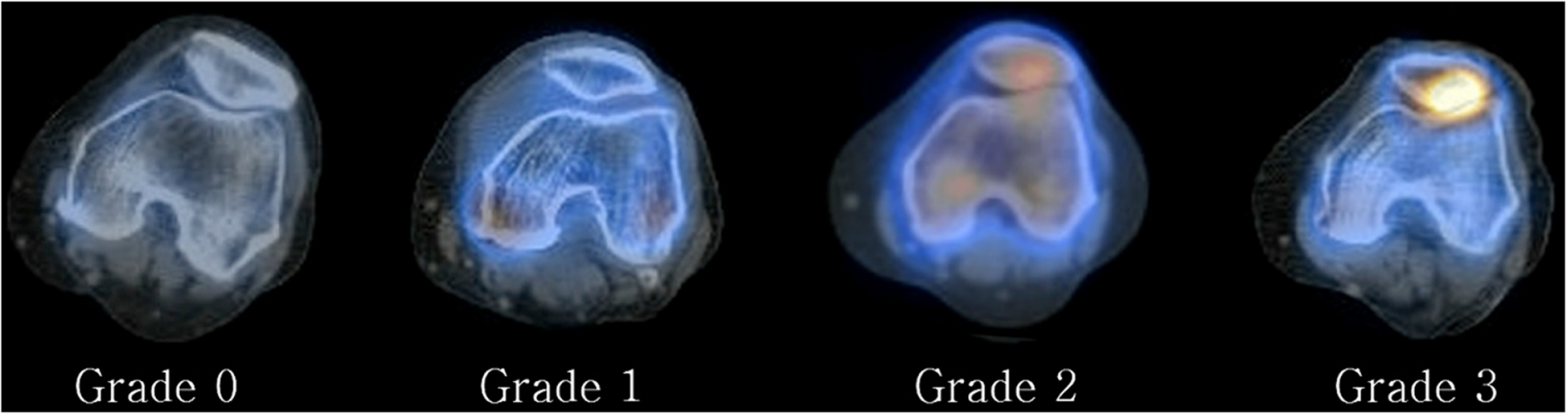
Single-photon emission computed tomography/computed tomography (SPECT/CT) uptake grading system for patella-femoral joint (patella grades shown). Grade 0 indicates no uptake (same as normal cancellous bone); grade 1, higher uptake than normal cancellous bone but lower than articular surface; grade 2, same uptake as articular surface; grade 3, higher uptake than articular surface

All of the 74 patients underwent SPECT-CT using the Infinia™ Hawkeye® 4 device (GE Healthcare, Milwaukee, WI, USA), which incorporates bone scintigraphy and a CT scan in a one-step procedure. Patients were injected with 1110 MBq (30 mCi) Tc-99 m hydroxymethylene diphosphonate (HDP), and images were taken 3 h post-injection. Multiplanar two-dimensional SPECT images were reconstructed in three phases (SPECT: 128 × 128 matrix; 32 frames; 35 s per frame; step and shoot). Multi-slice CT images (140 keV, 70–100 mA) of the entire lower extremity with multi-planar reconstruction (slice thickness: 1 mm; pitch and reconstruction interval: 1 mm) were also constructed. Scintigraphic uptake was graded on the following scale: grade 0, no uptake (equivalent to normal cancellous bone); grade 1, higher uptake than normal cancellous bone, but lower than articular surface; grade 2, same uptake as the articular surface; grade 3, higher uptake than the articular surface (Fig. 1). The femoral trochlea and patella were graded separately. SPECT-CT images were graded by two independent examiners, a knee specialist and a nuclear medicine specialist (two of the authors). Interobserver reliability of the assessment was evaluated using Cronbach ɑ; interobserver reliability was satisfactory (both the trochlea and patella had a Cronbach ɑ value of 0.79). Thus, assessments taken by one investigator (HYL, one of the authors) were used in the analyses.

### Patient management

After evaluation *via* SPECT-CT, each treatment protocol commenced on the next outpatient visit. The mean interval between SPECT-CT and commencement of treatment was 3 days. The treatment protocol included 8 weeks of activity modification (avoidance of kneeling, squatting, and the crossed-leg position), quadriceps strengthening exercises (straight leg raise, quad press, heel slide, and mini-squat), and nonsteroidal anti-inflammatory drugs (NSAIDs). Patients were re-evaluated at 4 weeks to determine compliance, and at 8 weeks to determine treatment response. They were classified into two groups: (1) responders (n = 40), whose knee condition improved by 8 weeks (no or minimal anterior knee pain during daily activities, corresponding to VAS ratings less than 3 of 10 points); and (2) non-responders (n = 34), whose knee condition did not improve (knee pain rated as 3 or more on the VAS) (Table 1). In non-responders, the cartilage condition of the PF joint was assessed by arthroscopy; all of the procedures were performed by a single surgeon (SBK). Arthroscopic assessment relied on the International Cartilage Repair Society (ICRS) clinical cartilage injury evaluation system [18]. One patient in the non-responder group refused arthroscopic evaluation; thus, 33 non-responding patients were assessed *via* arthroscopy.

**Table 1:** Comparison of demographic characteristics and four values for alignment of patellofemoral joint between the responder and non-responder groups

Parameter	Responders (n = 40)	Non-responders (n = 34)	*P*-value
Age (yr)	55 ± 6.4	53 ± 5.7	0.293
Gender (% female)	67.5 %	72.7 %	0.618
Body mass index (kg/m^2^)	26.1 ± 4.3	26.5 ± 4.5	0.762
Sulcus angle (°)	131 ± 7.4	128 ± 6.3	0.155
Congruence angle (°)	−5.7 ± 8.5	−2.8 ± 8.9	0.178
Insall-Salvati ratio	0.98 ± 0.14	0.96 ± 0.10	0.514
Q angle (°)	9 ± 3.1	11 ± 4.6	0.061

Data are presented as mean and standard deviation

### Statistical analysis

All of the statistical analyses were performed using the statistical package SPSS® 19.0.1 for Windows® (SPSS Inc., Chicago, IL, USA); *P* values < 0.05 were considered statistically significant. To determine the predictive value of SPECT-CT uptake for response to conservative management, we first compared median scintigraphic uptake grades of the patella and the femoral trochlea separately between the two groups. Then, we evaluated the proportion of patients with each uptake grade that did not respond to treatment (PPV for non-response) for both the patella and femoral trochlea. Fisher’s exact test was used to compare the number of non-responders in each uptake group. To examine the correlation of scintigraphic uptake and arthroscopically assessed focal chondral lesion severity in the PF joint among non-responders, we assessed agreement between the two values using Goodman and Kruskal’s gamma (γ) statistics. Gamma statistics were rated by the following criteria: γ > 0.81, very good agreement; 0.8 ≥ γ > 0.61, good agreement; 0.6 ≥ γ >0.41, moderate agreement; 0.4 ≥ γ >0.21, fair agreement; γ < 0.2, poor agreement.

## Results

**Fig. 2:**
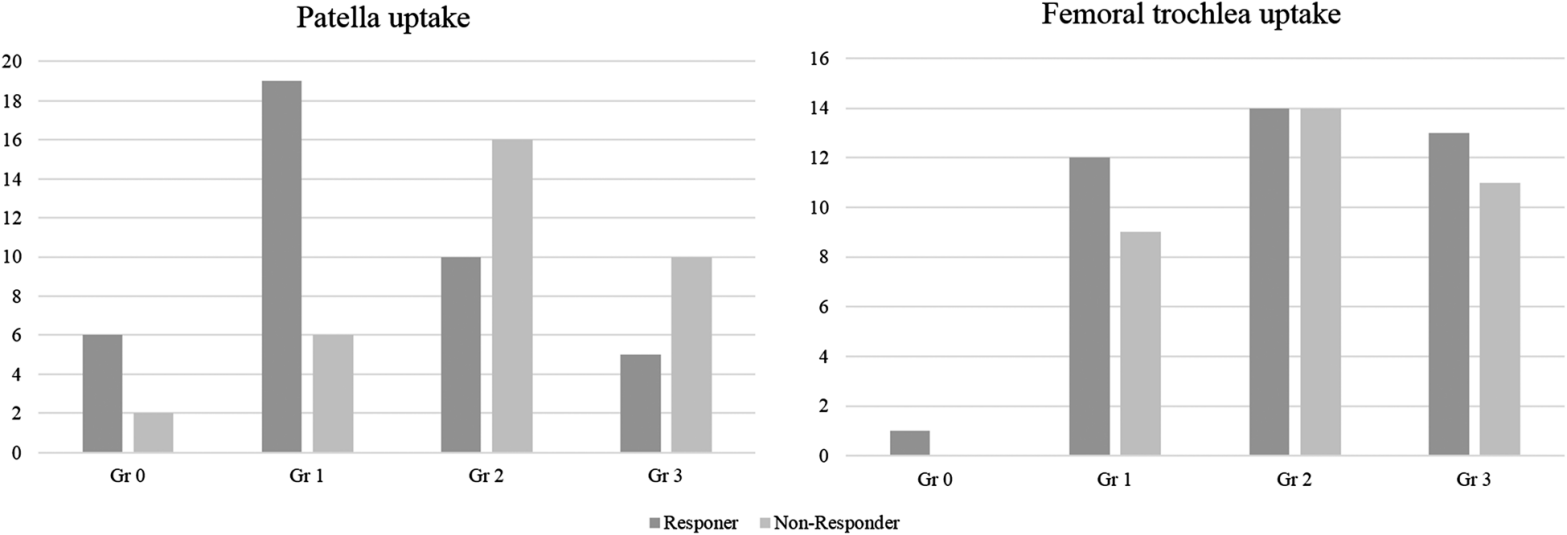
Uptake pattern according to response to therapy in the patella (p = 0.009) and femoral trochlea (p = 0.954)

No differences in mean age, gender distribution, BMI, or any radiological measurements were evident between responders and non-responders (Table 1). Scintigraphic uptake in the patella or femoral trochlea was at least grade 1 in all 74 patients. Median scintigraphic uptake in the patella was higher in non-responders than in responders (2 *vs.* 1). However, median scintigraphic uptake in the femoral trochlea did not differ between non-responders and responders (2 *vs.* 2) (Fig. 2). Patients with higher degrees of scintigraphic uptake in the patella were more likely to be non-responders (*P* = 0.009). The PPV of non-response for grade 2 or 3 patella uptake was 62 to 67 %. In contrast, only 24–25 % of patients with grade 0 or 1 patella uptake did not respond to treatment. However, the proportion of non-responders did not differ between patients with high and low uptake in the femoral trochlea (Table 2).

**Table 2:** Positive predictive value for non-response according to SPECT-CT grade

SPECT-CT grade	0	1	2	3	*p*-value^*^
PPV for non-response in the patella (non-responders/total patients)	25 % (2/8)	24 % (6/25)	62 % (16/26)	67 % (10/15)	**0.009**
PPV for non-response in the trochlea (non-responders/total patients)	0 % (0/1)	43 % (9/21)	50 % (14/28)	46 % (11/24)	0.954

^*^Fisher’s exact test, P-value with statistical significance (P < 0.05) is in bold

**Fig. 3:**
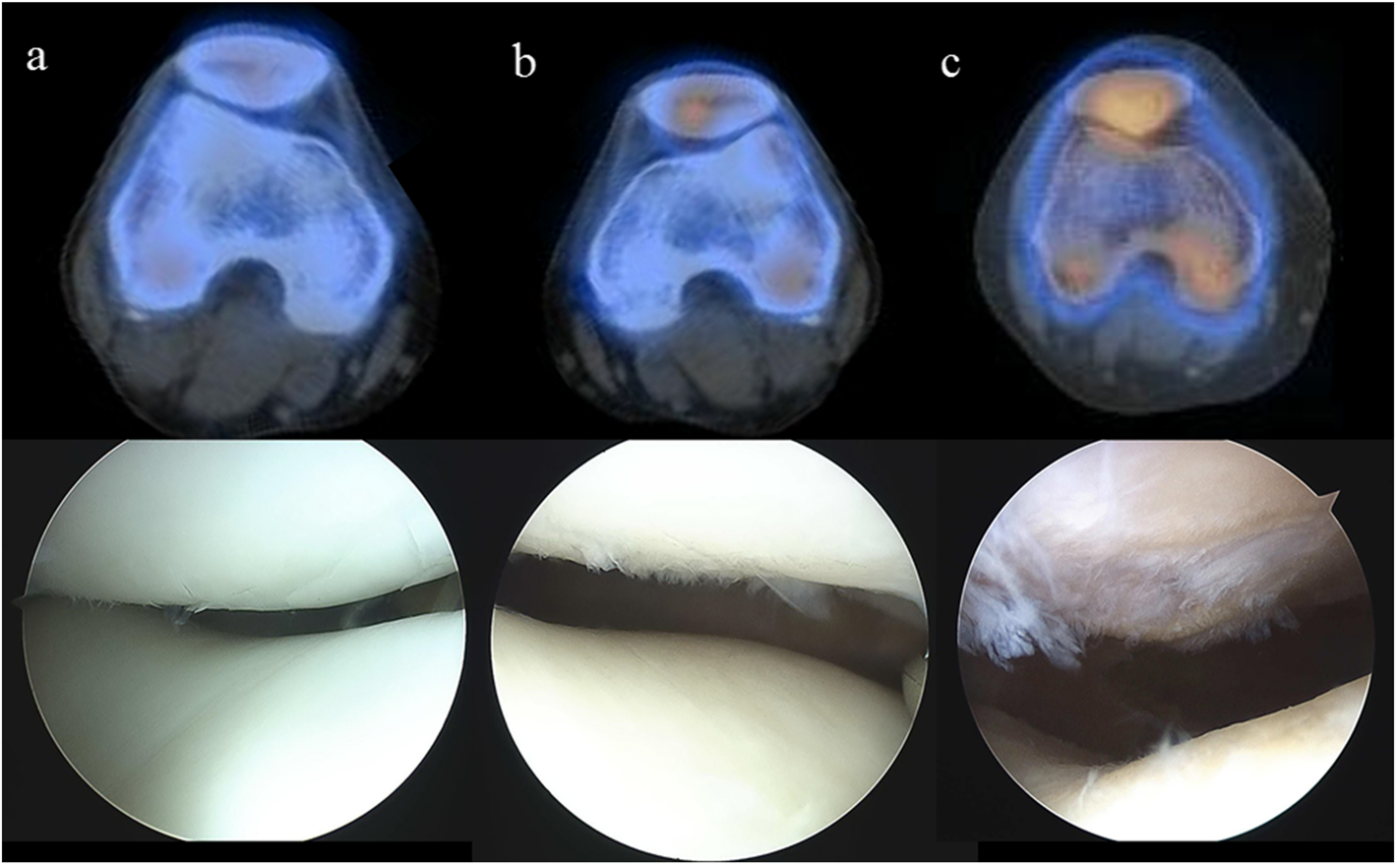
SPECT-CT image correlation with arthroscopic findings in the patella. **a** SPECT-CT grade 1 and ICRS grade 1; **b** SPECT-CT grade 2 and ICRS grade 2, **c** SPECT-CT grade 3 and ICRS grade 3

Among the 33 non-responders whose patellae were arthroscopically assessed, scintigraphic uptake correlated with the pathological grade of chondral lesions (Fig. 3). Arthroscopic assessment identified 3 cases of normal cartilage, 7 grade I lesions, 12 grade II lesions, 9 grade III lesions, and 2 grade IV lesions. Scintigraphic uptake and arthroscopic grades of chondral lesions in the patella agreed well (γ = 0.625; Table 3). Cartilage in the femoral trochlea was normal in two cases, whereas the remaining cases included 8 grade I cases, 11 grade II cases, 9 grade III cases, and 3 grade IV cases. Agreement between scintigraphic uptake and arthroscopic grade of chondral lesions in the trochlea was only fair (γ = 0.397; Table 4). Besides cartilage lesions, there were 3 cases of lateral meniscus tear, 2 of lateral partial discoid meniscus, and 8 of medial meniscus tear, which were concomitantly treated according to the indication.

**Table 3:** Single-photon emission computed tomography-computed tomography (SPECT-CT) and arthroscopic findings in 33 patellae (subject numbers are shown in each cell)

SPECT grade	Arthroscopic ICRS grade^1^					
	0	I	II	III	IV	Total
0	1	1	0	0	0	2
1	1	2	2	1	0	6
2	1	3	7	4	0	15
3	0	1	3	4	2	10
Total	3	7	12	9	2	33

Agreement between the two tests: ɣ = 0.625

^1^International Cartilage Repair Society (ICRS)

**Table 4:** Single-photon emission computed tomography-computed tomography (SPECT-CT) and arthroscopic findings in 33 femoral trochleae (subject numbers are shown in each cell)

SPECT grade	Arthroscopic ICRS grade^1^					
	0 (Negative)	I	II	III	IV	Total
0	0	0	0	0	0	0
1	1	1	5	2	0	9
2	1	5	5	2	0	13
3	0	2	1	5	3	11
Total	2	8	11	9	3	33

Agreement between the two tests: ɣ = 0. 397

^1^International Cartilage Repair Society (ICRS)

## Discussion

The most important finding of our study is that a higher degree of subchondral metabolism in the PF joint in patients with chronic anterior knee pain, particularly the patella, is predictive of a poorer response to conservative management. To date, this is the first study to show a relationship between SPECT-CT uptake and treatment response for chronic anterior knee pain.

Increased subchondral metabolism detected has been consistently observed by scintigraphy in anterior knee pain patients. Naslund et al. [14] found diffuse uptake in one or more of the bony compartments of the knee joint in approximately half of patients with anterior knee pain; Lorberboym et al. [19] concluded that SPECT is 100 % sensitive and 64 % specific in detecting patellofemoral lesions compared to arthroscopy. As subchondral bone metabolism increases with joint loading, SPECT allows the earlier detection of lesions than radiography or MRI. However, SPECT is limited by its lack of precise anatomical detail, which often leads to confusion, particularly in the PF joint [14, 19]. These drawbacks may be sidestepped by combining SPECT with CT, which provides high-resolution anatomical information.

SPECT-CT is increasingly recognized by the orthopedic field for its ability to identify changes in subchondral bone at a high spatial resolution [10, 12, 16, 20]. Hirschmann et al. [10] found that the intensity and distribution of SPECT-CT tracer uptake correlates with mechanical overload of the knee joint, leading the authors to advocate for its use in other orthopedic fields, such as osteoarthritis and arthroplasty [12, 21]. However, its value for the diagnosis and assessment of treatment response in chronic anterior knee pain had not yet been studied [14, 16]. Our results indicate that SPECT-CT can aid in establishing a diagnosis, and distinguishes patients who will respond to conservative treatment from those will not [3, 7, 22]. All of the patients with chronic anterior knee pain in our study had uptake greater than grade 1, reflecting increased subchondral bone metabolism.

Interestingly, patellar metabolic tracer uptake, and not trochlear uptake, was predictive of benefit from conservative management. We speculate that the higher shear stress in patellar cartilage than in the femoral trochlea during activity underlies this result [23]. Increased shear stress can increase the metabolic activity of subchondral bone, ultimately triggering anterior knee pain [12]. Schon et al. [17] reported that patella baja and increased patella tilt correlate with SPECT-CT uptake. Although we did not find such a correlation, our result also supports the conclusion that SPECT-CT uptake correlates with symptoms as well as anatomical findings.

Arthroscopic assessment of the 33 non-responders yielded a stronger correlation between scintigraphic uptake and pathological grade of chondral lesions in the patella. Our findings provide additional information on this issue: a more severe chondral lesion is more likely to lead to metabolic changes, which may be associated with a poorer prognosis. Although there is no widely accepted definition of early OA, altered subchondral bone metabolism with a higher grade chondral lesion and accompanying pain is a key feature of early stage OA [9, 24]. Taken together, SPECT-CT can benefit clinicians by predicting treatment response from conservative management in middle-aged patients with isolated atraumatic unilateral anterior knee pain.

Several limitations of the present study require consideration. First, because this study included only middle-aged patients, our findings may not be generalizable to patients in other age groups. However, we confined the study to middle-aged patients, because this patient group presents a significant diagnostic challenge in clinical practice. Second, because the optimal treatment method for chronic anterior knee pain is controversial, it may be debatable whether our conservative management was adequate. However, we applied an 8-week program of comprehensive conservative management, including activity modification, muscle strengthening exercise, and use of NSAIDs; in addition, we determined compliance with management at four weeks. Thus, we believe that this program is adequate to determine responsiveness to conservative management of chronic anterior knee pain. Third, the present study did not involve a control group for investigation of correlation between metabolic tracer uptake and arthroscopic grades, as we did not evaluate the knees of responders arthroscopically. However, because such an invasive procedure is unethical in the absence of a clinical problem, results from this group would be less meaningful in practice. Fourth, outcomes after arthroscopic surgery were not presented. Although our preliminary results reveal the effectiveness of arthroscopic procedures in non-responders, we believe that inclusion of clinical results after surgery is beyond the goals of this study and might create redundancy. We hope to address this issue in the future by assessing longer term follow-up results. Fifth, we did not conduct predictive modeling; future studies are required to confirm the potential of SPECT-CT for predicting the clinical response to conservative therapy. Sixth, we did not use absolute VAS values, rather dichotomizing responder and non-responders using a VAS pain score cutoff of 3. Although the use of absolute VAS scores would have yielded more information, we found that VAS pain scores varied widely over time. Thus, we considered that use of a cutoff average VAS score for each patient would allow us to more reliably distinguish responders from non-responders.

## Conclusions

Patients with greater metabolic tracer uptake in the PF joint on SPECT-CT, particularly in the patella, are less likely to respond to conservative management; furthermore, patella uptake correlates with arthroscopically assessed grading of patellar chondral lesions. These findings suggest that SPECT-CT can aid clinicians in predicting treatment response from conservative management in middle-aged patients with isolated atraumatic unilateral anterior knee pain. In addition, the findings suggest that more aggressive treatment should be considered for patients with high patellar uptake on SPECT-CT.

## Abbreviations

AKP: Anterior knee pain
AP: Anteroposterior
CRP: C-reactive protein
ESR: Erythrocyte sedimentation rate
HDP: Hydroxymethylene diphosphonate
MRI: Magnetic resonance imaging
NSAIDs: Nonsteroidal anti-inflammatory drugs
OA: Knee osteoarthritis
PF: Patellofemoral
PPV: Positive predictive value
SPECT-CT: Single-photon emission computed tomography-computed tomography
TF: Tibiofemoral
